# Personality Disorders in Obsessive-Compulsive Disorder: A Comparative Study versus Other Anxiety Disorders

**DOI:** 10.1155/2013/856846

**Published:** 2013-12-21

**Authors:** Josep Pena-Garijo, Silvia Edo Villamón, Amanda Meliá de Alba, M. Ángeles Ruipérez

**Affiliations:** ^1^Department of Basic and Clinical Psychology and Psychobiology, Jaume I University of Castelló, Sos Baynat Avenue, 12071 Castelló, Spain; ^2^Department of Mental Health, General Hospital of Castelló, 13 Benicassim Avenue, 12004 Castelló, Spain; ^3^Hospital de Día, Provincial Hospital of Castelló, Cardenal Costa 10, 12004 Castelló, Spain; ^4^Department of Educational and Developmental Psychology, University of Valencia, 21 Blasco Ibañez Avenue, 46010 Valencia, Spain

## Abstract

*Objective*. The purpose of this paper is to provide evidence for the relationship between personality disorders (PDs), obsessive compulsive disorder (OCD), and other anxiety disorders different from OCD (non-OCD) symptomatology. *Method*. The sample consisted of a group of 122 individuals divided into three groups (41 OCD; 40 non-OCD, and 41 controls) matched by sex, age, and educational level. All the individuals answered the IPDE questionnaire and were evaluated by means of the SCID-I and SCID-II interviews. *Results*. Patients with OCD and non-OCD present a higher presence of PD. There was an increase in cluster C diagnoses in both groups, with no statistically significant differences between them. *Conclusions*. Presenting anxiety disorder seems to cause a specific vulnerability for PD. Most of the PDs that were presented belonged to cluster C. Obsessive Compulsive Personality Disorder (OCPD) is the most common among OCD. However, it does not occur more frequently among OCD patients than among other anxious patients, which does not confirm the continuum between obsessive personality and OCD. Implications for categorical and dimensional diagnoses are discussed.

## 1. Introduction

Over the last few years the study of Obsessive Compulsive Disorder (OCD) has raised an increasing amount of interest in different fields. As its name suggests, the clinical condition of OCD is characterised by the presence of obsessions or compulsions. An obsession is a recurring and persistent thought, idea, or image, which is experienced in a parasitic way and its content is usually undesirable and produces anxiety; to a large extent it is involuntary and disturbs the course of the subject's normal thought activity. Sometimes it is accompanied by the need to perform a certain action (either behaviour or other thoughts), which is performed as a compulsion or obsessive ritual with the aim of reducing the feeling of distress.

The term “obsessive” has been used to refer to symptoms or personality traits, such as aspects related to the previous personality of the individual; the form the disorder takes as it develops; the possibility of confusing a symptom and a trait when the symptom lasts for years; or the transformation of a symptom into a trait when the individual accepts and includes it as a way of adapting to a painful and invalidating illness. All these questions are relevant in the study of personality and its relation with Axis I disorders.

A topic of debate in the study of this pathology has been the continuum between obsessive personality and OCD. According to Vallejo [1], previous works (generally about psychoanalytic orientation) indicate the existence of obsessive personalities in obsessive patients (50–80%), which would speak in favour of such a continuum. However, recent studies using DSM-based criteria point to lower percentages—a finding that queries the existence of a continuum between obsessive personalities and OCD. In studies based on DSM-III-R criteria [2] the relation that is found is not specifically between OCD and Obsessive Compulsive Personality Disorder (OCPD). Nevertheless, cluster C is, in general, the one that is most frequently associated with OCD [3, 4]. Albert et al. [5] analysed 15 studies conducted between 1999 and 2002, in which the presence of OCPD was measured in samples of patients with OCD. Results showed a great variability in comorbidity measures (3–36%) and only a minority of OCD patients (18%) also met criteria for OCPD.

The same results were found in a study carried out by Torres et al. [6] using data from the *2000 British National Survey of Psychiatric Morbidity* [7, 8]. In this study they evaluated the prevalence of personality disorders in OCD adults in a general population sample, the differences in pathological personality between genders, comorbidity with other anxiety disorders, and the presence of obsessions, compulsions, or both. Results indicated that OCD patients show more PD in general in comparison to other groups with other anxiety disorders, whereas PD from cluster C were the most common among OCD.

Given that categorical proposals still prevail from the consecutive editions of the DSM [9] and that in the clinical field the comprehension of these disorders from categorical proposals is common, the main purpose of this paper is to provide evidence for the continuum between OCD and personality disorders. In order to carry out this purpose, two secondary objectives were planned: first, symptomatology of PD was analysed in three groups with different severity gradient. Second, differences in frequency of categorical PD between two clinical groups were analysed, paying special attention to the frequency of OCPD in both clinical groups.

## 2. Material and Methods

### 2.1. Description of the Sample

Demographic and clinical data are summarised in Table 1. The final sample consisted of a group of 122 participants, who were divided into three groups (41 OCD, 40 non-OCD anxiety, and 41 control group). Clinical samples were obtained from patients treated at the outpatients' service of the Department of Mental Health 2 at the General Hospital of Castelló (Spain). The control group was composed of university students and students from an adult school in the same city. At the beginning of the study all the participants were told of the purpose of the research, and those who volunteered to participate then signed an informed consent form. As shown in Table 1 there were no significant statistical differences between the three groups in the demographic variables.

Regarding depressive symptoms assessed through BDI-II, significant differences between control group and both clinical groups, OCD group and non-OCD group, were found after correcting by Bonferroni's test (*P* = .000). However, differences between clinical groups did not rise significant level (*P* = .657).

More specifically, patients with OCD obtained scores within moderate range in Y-BOCS (mean = 24.93, Sd = 6.47).

Participants in the study were assessed according to DSM-IV-TR criteria. In relation to Axis I, all participants in OCD group met criteria for a primary diagnosis of OCD, and only 17 showed a secondary diagnosis. The non-OCD group met criteria for a primary diagnosis of anxiety disorder with the exception OCD, and 18 participants showed another secondary diagnosis. In relation to the Axis II, Table 2 shows in more detail diagnosis of PD in both clinical groups (OCD and non-OCD). Finally, control group did not meet criteria for any disorder neither in Axis I nor Axis II.

### 2.2. Measures

International Personality Disorder Examination [10] (IPDE), Spanish version by Lopez-Ibor et al. [11], is a diagnostic tool composed of a self-report and a semistructured clinical interview used to evaluate the different PDs according to DSM-IV [9] and/or ICD-10 criteria [12]. Only the self-report was used as a screening measure. The choice of this instrument is justified because of its widespread use in both the clinical and research fields, together with its positive psychometric properties, that is, average kappa coefficient 0.73 and test-retest 0.87 [13].

Structured Clinical Interview for DSM-IV Axis I Disorders [14] (SCID-I), semi-structured interview used for evaluating some of the clinical symptoms described in DSM-IV in Axis I. It correctly evaluates affective disorders, schizophrenia and other psychotic disorders, such as substance-related disorders, anxiety disorders, somatomorphic disorders, eating disorders, and adaptive disorders.

Structured Clinical Interview for DSM-IV Axis II Personality Disorders [15] (SCID-II) is a semi-structured interview used for evaluating different PDs described in the DSM-IV from the categorical approach to determine the actual diagnosis. Moreover, each question has four possible answers to choose from, which also allows a dimensional approach.

Yale-Brown Obsessive Compulsive Scale (Y-BOCS) [16, 17], Spanish version by Sal et al. [18]. is a semi-structured interview enabling the clinician to establish an overall severity rating as well as separate severity ratings for both obsessions and compulsions. The severity scale of the Y-BOCS contains 10 items: 5 for obsessions and 5 for compulsions. Satisfactory reliability, and validity have been reported for the Y-BOCS [19].

Beck Depression Inventory (Second Edition) (BDI-II) [20], Spanish version by Sanz et al. [21], is a self-administered tool for screening and assessing the severity of depression in adolescents and adults. Twenty-one items assess the intensity of depression in diagnosed patients as well as detect possible depression in normal population. The BDI-II has been shown to be a reliable and well-validated measure of depressive symptomatology [21].

### 2.3. Evaluation Procedure

All clinical patients were recruited from public mental health services (Department 2 of Health in Valencian Region of Spain) through different procedures as follows. In a three years period, all first referral patients who met criteria for OCD were recruited for the OCD group. In order to equal the groups (on gender, age, and instruction level), non-OCD group and control group were recruited after OCD group was conformed. In the same mental health service, participants of the non-OCD group were selected from patients in treatment whose primary diagnosis was anxiety disorder different from OCD. The control group was composed of university students and students from an adult school in the same city.

After agreeing to participate in the study, all subjects were evaluated by an independent clinician using the SCID-I, in order to guarantee the absence/presence of pathology in Axis I, based on the criteria established before. Given the comorbidity of depressive symptomatology in anxiety disorders, BDI-II was administered. To provide an index of the severity of current obsessions and compulsions, subjects with an OCD diagnosis answered Y-BOCS.

Finally, in order to assess pathology in Axis II, IPDE questionnaire and SCID-II were administered.

### 2.4. Statistical Considerations

Data were analysed using the statistical pack SPSS version 17. According to the two secondary objectives proposed in this study, two different analyses were performed. First, IPDE dimensional scores of the three groups were compared with a one-way ANOVA with Bonferroni correction. For the second objective, to compare categorical personality disorders frequencies, ANOVA analysis was used.

## 3. Results

The first analysis conducted did not reveal any significant differences between clinical groups in any of the contrasts. However, significant differences arose when control and non-OCD group were compared for all PDs, with the exception of comparisons on schizoid and histrionic PDs. On the other hand, scores of OCD group showed significantly higher scores when compared to control group in all PDs but paranoid and histrionic PDs (see Table 2).

Analysis for the second objective only showed significant differences between clinical groups on the frequency of accumulative disorders of cluster A and cluster C as shown in Table 3.

## 4. Discussion

The aim of this study was to provide evidence for the continuum between OCD and personality disorders by analysing symptomatology of PD in three groups with different severity gradient, and by studying the frequency of categorical PD between two clinical groups with anxiety disorders (OCD and non-OCD).

Results in our study confirmed the higher prevalence rate of personality disorders in OCD patients with regard to the general population but also confirmed the higher rate of PD in other anxiety disorders which were phenomenologically well characterized and different from OCD. Existence of a diagnosis of PD in Axis II in both clinical groups ranges from 40% to 50%, OCD and non-OCD. Although in our study the differences between the two clinical groups were not significant, an upward trend towards patients with a diagnosis of OCD was found. These results point out a high percentage of comorbidity between anxiety disorders and pathology in Axis II, as previous studies have shown [22–29]. In line with Torres et al.'s study [6], rate of positives in screening for PD was 78% in a group of OCD patients (*n* = 108). The most frequent diagnoses were paranoid, avoidant, schizoid, and schizotypal, which is a result that follows the same line as our study. This could mean that among individuals with a diagnosis of OCD, pathological personality prevails over other neuroses and over the general population.

Noteworthy conclusions are brought by analysing differences by PD clusters. Around 10% of the cases from the obsessive group present diagnoses from cluster A in comparison to the absence of these diagnoses in the non-OCD anxiety group; these results agree with those obtained in previous studies [27, 30, 31]. The same studies also found an increase in cluster B pathologies, whereas our data indicate that the non-OCD anxiety group is the one that obtains the most diagnoses from that cluster, with an absence of positive cases in the OCD group. In this study, while dimensional measures (i.e., IPDE) are used, anxiety disorders presence in Axis I entail a higher presence of PD related to cluster C, which is sustained by close to moderate effect size, in contrast to small effect size in cluster A and B. These results obtained with dimensional instruments gain further strength when they coincide with categorical instrument findings, given that both clinical groups had a higher diagnostic presence in Axis II.

The impact of anxiety disorder presence is also found in relation to cluster C when categorical measures are used. Moreover, when categorical criteria are applied through structured clinical interviews (SCID-I and SCID-II), the screening done through IPDE demonstrates adequate discriminant power between clinical and nonclinical subjects.

In other words, it seems obvious that both clinical groups are more vulnerable to PD in cluster C. This has already been demonstrated in previously cited studies that agree with the definition of this group in the DSM IV-TR, where they are called “anxious-frightened” people. Additionally, relation of cluster C and OCD has been reported in recent exploratory, genetic, and familiality studies [32–34]. These results point out that there is a higher degree of personality pathology among patients than among non-pathology individuals, although no specific PD is found between clinical groups.

Nowadays, the use of categorical versus dimensional instruments in clinical practice is still controversial. Differences in results between categorical and dimensional assessments highlight the importance of the selection process of diagnosis instrument and its impact on results interpretation. Self-reports like IPDE entail that patients self-identify more pathology than clinicians would do through interviews. This phenomenon could be due to method effect, where patients before a questionnaire try to answer every item looking for some grade of applicability of it on them, avoiding answering “never,” even when the item describes extreme scenarios. That causes a trend of subtle presence of pathology even when there is not. On the other hand, categorical assessment developed through clinical interview provides more reliable information of the patient. This is why we consider it especially important that in research both dimensional and categorical assessment should be practiced with a double purpose: first, by means of screening instruments, clinical or general population sample can easily be selected; and second, in order to clarify in more detail the pathology presented in those cases, were suspicions arise from the screening procedure, assessment should be completed with clinical interviews.

It is worth mentioning that OCPD was the most frequent disorder found in the two clinical groups, followed by avoidant type. However, when examining the association between OCD and OCPD, our results did not confirm a specific relationship given that no significant differences were found between clinical groups. Results in this study fail to demonstrate the alleged continuum between both disorders [32, 35], contradicting recent studies that have suggested comorbidity between OCD and OCPD as a distinct subtype of OCD and describe its characteristics [36, 37].

The study limitations should be pointed, such as the sample size, which is rather small to be able to draw epidemiological conclusions. This could be explained in part by the recruitment procedure used. It is important to highlight that patients with OCD were recruited from outpatients consecutively referred to the Department of Mental Health 2 at the General Hospital of Castelló (Spain) in a three years period, which reflects disorder prevalence described in DSM-IV-TR. This sampling method did not facilitated to obtain information about age of onset and course of symptom dimensions. Additionally, treatment factors were not controlled, and thus it is possible that the treatment history affected the results of our one-point evaluation study. From a translational approach [38], based on recent research which highlighted that early age of onset could be a marker of symptom severity [39–41], mental health services should seriously consider establishing standard evaluation protocols in which this information is compiled, bearing in mind continuities and discontinuities in psychopathology between childhood and adult life.

Similarly, taking into account the type of statistical analysis carried out, causality relationships between pathology described in Axis I and II cannot be concluded in any direction. Furthermore, additional information regarding the development of clinical conditions is needed in order to establish whether or not relationship between Axis I and Axis II is the result of a long-term adaptation of the patient to the disease. Two decades ago, Baer and Jenike [42] proposed an interesting hypothesis that claimed that, in some cases, OCPD was secondary to OCD and was a way of adapting to the behaviours and the lifestyle that this marks. At this point it is worth remembering Tyrer's warning regarding the risk of confusion that can arise from the fact that phobic and obsessive symptoms, when manifested for many years, could end up satisfying trait criteria and being considered representative of a premorbid personality without this actually being the case [43].

Frequently in research, depressive symptomatology and gender mask PD frequency in patients with OCD. Taking into account that no significant differences were found between both clinical groups in gender and depressive symptomatology, both could be excluded as possible moderators of results. Controlling for these variables allowed effectively isolate comorbidity effect between different types of anxiety disorders and PD.

## 5. Conclusions

This study indicates that obsessive compulsive patients show a high rate of pathology in Axis II that is higher than in the non-OCD anxiety patients. The majority of the PDs that obsessive patients show corresponds to cluster C, like the rest of the patients with anxiety disorders. OCPD is the most common PD in both groups, although it is not more common in the OCD than in the other anxiety disorder groups. Meeting diagnostic criteria for PD in OCD may be a marker of severe symptomatology in OCD [44]. Future research should examine personality traits in OCD patients, assessed by normal and pathological scales, in order to discern possible personality profiles related to OCD subtypes [45, 46]. The existence of a continuum between OCDP and OCD is not confirmed.

## Figures and Tables

**Table 1:** Demographic characteristics and BDI-II results of subjects with OCD, without OCD, and control subjects^a^.

	OCD (*n* = 41)	Non-OCD (*n *= 40)	Control (*n *= 41)	Test statistic	df	*P*	ES^b^
Age	35.78 (10.63)	35.37 (10.14)	34.90 (9.7)	*F* = 0.08	2,118	.928	.001
Gender (% male)	16 (39.02)	12 (30)	16 (39.02)	*χ* ^2^ = 0.95	2	.622	.088
BDI-II	20.07 (12.02)	23.28 (14.80)	7.80 (6.79)	*F* = 19.97	2,119	.000	.251
Educational level				*χ* ^2^ = 0.95	6	.911	.132
No studies	1	1	1				
Primary studies	12	15	12				
Secondary studies	12	12	15				
University studies	16	12	13				

^a^Results by group are presented as *n* (% of subjects) for *χ* ^2^ analyses and mean (SD) for ANOVA.

^b^Effect sizes are presented as phi for *χ* ^2^ analyses and eta for ANOVA (0.10 = small, 0.30 = medium, 0.50 = large for both effect size measures).

**Table 2:** Results of ANOVA analysis for comparisons among OCD, non-OCD, and control group in IPDE scores.

	OCD (*n* = 41)		Non-OCD (*n *= 40)		Control (*n *= 41)		*F* (2,119)	*P*	*η* ^2^	Post hoc
	M	SD	M	SD	M	SD				
Paranoid	2.17	1.58	2.48	1.66	1.59	1.12	3.83	.024	.061	OCD = non-OCD OCD = control Non-OCD > control
SCHIZOID	2.98	1.72	2.43	1.50	1.68	1.29	7.50	.001	.112	OCD = non-OCD OCD > control Non-OCD = control
SCHIZOTYP.	2.63	2.08	2.25	1.41	1.07	1.31	10.08	.000	.145	OCD = non-OCD OCD > control Non-OCD > control
Total cluster A	7.78	3.93	7.15	3.77	4.34	2.95	10.74^a^	.000	.153	OCD = non-OCD OCD > control Non-OCD > control
Borderline	3.68	2.05	4.5	1.78	2.05	1.95	17.23	.000	.225	OCD = non-OCD OCD > control Non-OCD > control
Antisocial	0.68	0.88	0.85	1.03	0.78	1.13	0.28	.759	.005	—
Narcissist	2.31	1.57	2.30	1.49	2.24	1.46	.026	.974	.000	—
Histrionic	2.66	1.58	3.44	1.82	2.51	1.73	3.34	.039	.054	OCD = non-OCD OCD = control Non-OCD = control
Total cluster B	9.34	3.90	11.23	4.25	7.59	4.80	7.08^a^	.001	.107	OCD = non-OCD OCD > control Non-OCD > control
Avoidant	4.68	2.21	4.15	2.35	2.83	2.04	7.71	.001	.115	OCD = non-OCD OCD > control Non-OCD > control
Dependent	2.90	2.11	3.38	1.98	1.46	1.61	11.03	.000	.156	OCD = non-OCD OCD > control Non-OCD > control
Obsessive	3.61	1.97	4.10	1.79	2.15	1.48	13.57	.000	186	OCD = non-OCD OCD > control Non-OCD > control
Total cluster C	11.20	5.04	11.63	4.32	6.44	3.8	17.33^a^	.000	.226	OCD = non-OCD OCD > control Non-OCD > control

Note: ^a^degrees of freedom (2,118).

**Table 3:** PD comorbidity in clinical subjects with OCD and without OCD.

	OCD (*n* = 41)		Non-OCD (*n* = 40)		*χ* ^2^ (1)	*P*	*φ*
	*N*	%	*N*	%			
Paranoid	1	2.4	0	0	0.99	.320	−.110
Schizoid	1	2.4	0	0	0.99	.320	−.110
Schizotypal	2	4.9	0	0	2.00	.157	−.157
Total cluster A	4	9.8	0	0	4.12	.043	−.225
Borderline	0	0	1	2.5	1.04	.308	.113
Antisocial	0	0	0	0	—	—	—
Narcissistic	0	0	0	0	—	—	—
Histrionic	0	0	2	5	2.10	.147	.161
Total cluster B	0	0	3	7.5	3.19	.074	.199
Avoidant	7	17.0	3	7.5	1.72	.190	.190
Dependent	4	9.8	1	2.5	1.84	.175	−.151
Obsessive	10	24.4	7	17.5	0.58	.446	−.085
Total cluster C	21	51.2	11	27.5	4.77	.041	.029
